# The modulation of immune cell death in connection to microRNAs and natural products

**DOI:** 10.3389/fimmu.2024.1425602

**Published:** 2024-12-20

**Authors:** Ya-Ting Chuang, Ching-Yu Yen, Jen-Yang Tang, Fang-Rong Chang, Yi-Hong Tsai, Kuo-Chuan Wu, Tsu-Ming Chien, Hsueh-Wei Chang

**Affiliations:** ^1^ Department of Biomedical Science and Environmental Biology, PhD Program in Life Sciences, College of Life Science, Kaohsiung Medical University, Kaohsiung, Taiwan; ^2^ School of Dentistry, Taipei Medical University, Taipei, Taiwan; ^3^ Department of Oral and Maxillofacial Surgery, Chi-Mei Medical Center, Tainan, Taiwan; ^4^ School of Post-Baccalaureate Medicine, Kaohsiung Medical University, Kaohsiung, Taiwan; ^5^ Department of Radiation Oncology, Kaohsiung Medical University Hospital, Kaohsiung Medical University, Kaohsiung, Taiwan; ^6^ Graduate Institute of Natural Products, Kaohsiung Medical University, Kaohsiung, Taiwan; ^7^ Department of Pharmacy and Master Program, College of Pharmacy and Health Care, Tajen University, Pingtung, Taiwan; ^8^ Department of Computer Science and Information Engineering, National Pingtung University, Pingtung, Taiwan; ^9^ Department of Urology, Kaohsiung Medical University Hospital, Kaohsiung, Taiwan; ^10^ Department of Urology, Kaohsiung Gangshan Hospital, Kaohsiung Medical University, Kaohsiung, Taiwan; ^11^ Center for Cancer Research, Kaohsiung Medical University, Kaohsiung, Taiwan; ^12^ Department of Medical Research, Kaohsiung Medical University Hospital, Kaohsiung, Taiwan

**Keywords:** ICD, DAMPs, cytokines, microRNAs, natural products, targets

## Abstract

Immunogenic cell death (ICD) spatiotemporally regulates damage-associated molecular patterns (DAMPs) derived from dying cancer cells to signal the immune response. Intriguingly, these DAMPs and cytokines also induce cellular responses in non-immune cells, particularly cancer cells. Several ICD-modulating natural products and miRNAs have been reported to regulate the DAMP, cytokine, and cell death responses, but they lack systemic organization and connection. This review summarizes the impacts of natural products and miRNAs on the DAMP and cytokine responses and cancer cell death responses (apoptosis, autophagy, ferroptosis, necroptosis, and pyroptosis). We establish the rationale that ICD inducers of natural products have modulating effects on miRNAs, targeting DAMPs and cytokines for immune and cancer cell death responses. In conclusion, DAMP, cytokine, and cell death responses are intricately linked in cancer cells, and they are influenced by ICD-modulating natural products and miRNAs.

## 1 Introduction

### 1.1 Immunogenic cell death, damage-associated molecular patterns, and cytokines

ICD induces antitumor immunity by triggering several immune signals and damage-associated molecular patterns (DAMPs) involved in immune and cell death responses (1). DAMPs are molecules that induce intracellular responses but generate immunogenic responses in an extracellular environment (2). Generally, DAMPs are released by damaged or dying cells and tissues, triggering an innate immune response against these damaged and infected cells as well as cancer cells (3).

The danger signals from DAMPs include the cell surface exposure of calreticulin (CALR), the release of the high-mobility group box 1 (HMGB1) protein, and the secretion of ATP (4). Subsequently, they cooperatively activate dendritic cells and cytotoxic T lymphocytes (CTLs), causing them to kill cancer cells (5). LDL receptor-related protein-1 (CD91), P2X7 receptor (P2X7R), and Toll-like receptor 2 (TLR2) are located on the dendritic cell surface and recognize CALR, ATP, and HMGB1, respectively.

In addition to DAMPs, ICD inducers also promote the secretion of inflammatory cytokines from cancer cells. The DAMPs and cytokines activate dendritic and natural killer (NK) cells, promoting the secretion of effector cytokines. After ICD inducer treatment, the dying cancer cells promote DAMP responses, and, in turn, they release cytokines that activate the immune response, such as C-X-C motif chemokine ligand 10 (CXCL10; IL8) and interleukin 6 (IL6) (6, 7); additionally, IFNG (IFN-γ) is released by T helper 1 (Th1) cells and CTLs and interleukin 17A (IL17A; IL17) is released by Th17 cells (8). Moreover, activated dendritic cells secrete interleukin 12 (IL12), which causes NK cells to secrete interferon γ (IFNG; IFNγ) and tumor necrosis factor-alpha (TNF; TNFA; TNFα) (9). Furthermore, macrophages secrete interferon β (IFNB1; IFNβ1; IFNB), promoting apoptosis in neutrophils (10). Myeloid-derived suppressor cells (MDSCs) are responsible for the immune suppression activity of macrophages and dendritic cells (11). Therefore, ICD modulates DAMP and cytokine responses.

### 1.2 Some DAMPs and cytokines involved in ICD were selected as ICD gene candidates

As described above, ICD initiates spatiotemporal DAMP signals, such as the cell surface translocation of CALR and heat shock proteins (HSP70 and HSP90) and the release of ATP and HMGB1, leading to cell death (12). Consequently, DAMPs and cytokines are vital for ICD induction.

**Figure 1:**
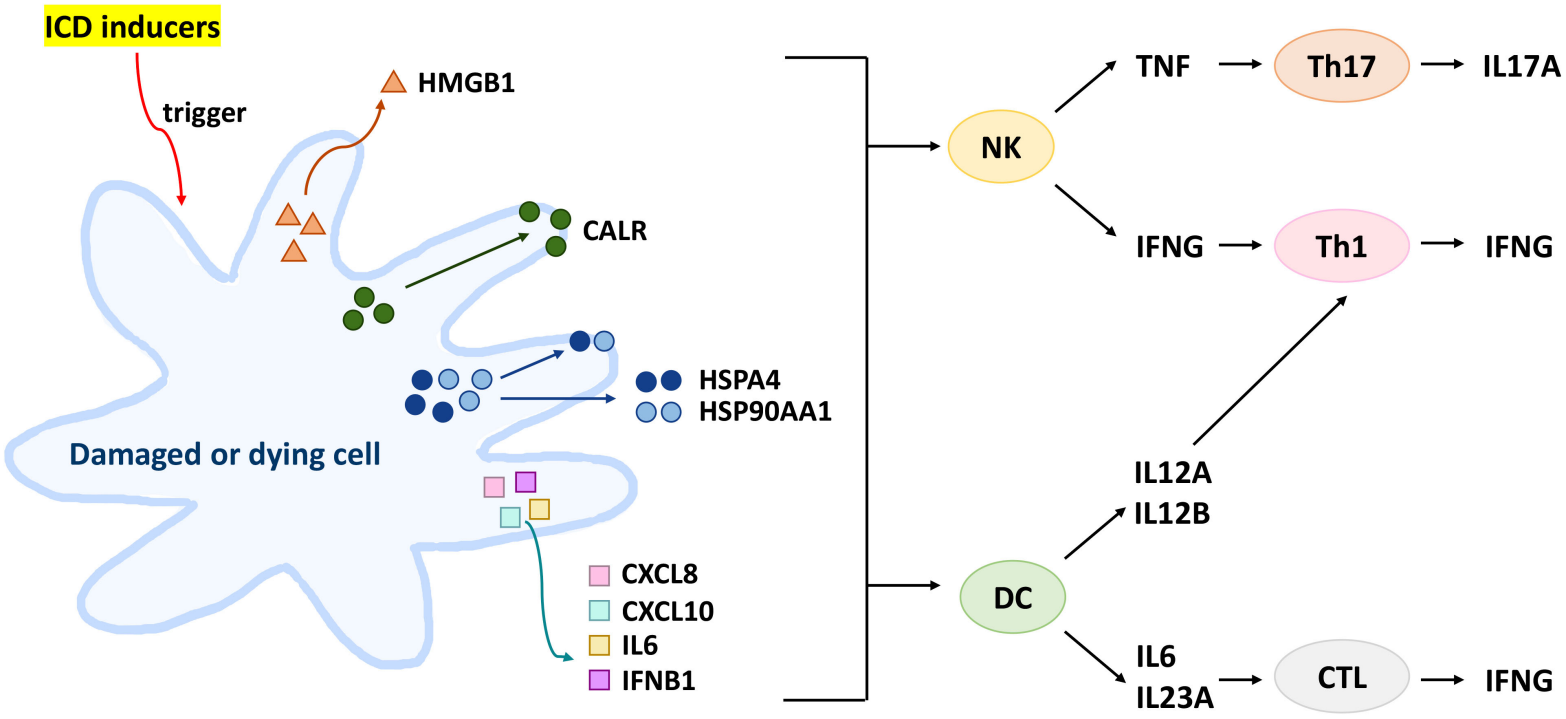
Protein signaling pathway involving both DAMPs and selected cytokines in ICD modulation. ICD inducer triggers HMGB1 secretion, CALR surface exposure, HSPA4/HSP90AA1 surface exposure and secretion, and inflammatory cytokine secretion from dying cancer cells or damaged cells. Subsequently, HSPA4/HSP90AA1 and inflammatory cytokines can activate both NK and DC cells. NK cells secrete INFG and TNF to activate Th1 and Th17 to secrete IFNG and IL17A, respectively. Moreover, DC cells secrete IL12A and IL12B to activate Th1 cells and secrete IL6 and IL23A to activate CTL cells for IFNG secretion.

In this review, we selected DAMPs involved in ICD modulation, including HMGB1, CALR, heat shock protein family A (HSP70) member 1A (HSPA1A), HSP70 member 1B (HSPA1B), heat shock protein 90 alpha family class A member 1 (HSP90AA1), and heat shock protein 90 beta family member 1 (HSP90B1) (1, 3, 12, 13). The selected cytokines involved in ICD modulation include interleukin 6 (IL6), C-X-C motif chemokine ligand 8 (CXCL8; IL8), CXCL10, IL12A, IL12B, IL17A, IL23A (IL23), IFNB1, IFNG, and TNF (14, 15). The protein signaling pathway involving both DAMPs and selected cytokines in ICD modulation were shown (**Figure 1**) (15–21).

### 1.3 miRNAs have ICD-modulating effects

miRNAs are short nucleotide (21-23 nts) molecules that can regulate genes both positively (22–25) and negatively (2). Several miRNAs exhibit tumor-promoting or suppressing effects. In the immune response, miRNAs may regulate the ICD-modulated expression, translocation, and secretion of some DAMPs (2). Moreover, miRNAs may regulate cytokine responses in a manner that modulates the immune response (26, 27). Consequently, DAMP-targeting miRNAs are potential regulators of immune-modulated responses and cell death in cancer cells (2).

### 1.4 ICD inducers of natural products

Drugs with the ability to induce ICD have anticancer effects by enhancing DAMP and cell death responses (28). Several natural products exhibiting ICD-inducing functions have been reported (28). However, the impact of miRNA regulation on natural ICD-modulating products remains unclear. Later, we will illustrate the organization between ICD, natural products, and miRNAs in detail.

### 1.5 Rationale for this review

Many natural products exhibit miRNA-modulating effects when used for anticancer treatment (29, 30). Moreover, many of these products have induced ICD (31, 32). Accordingly, natural products have the potential to regulate ICD through miRNAs, but they lack systemic organization.

**Figure 2:**
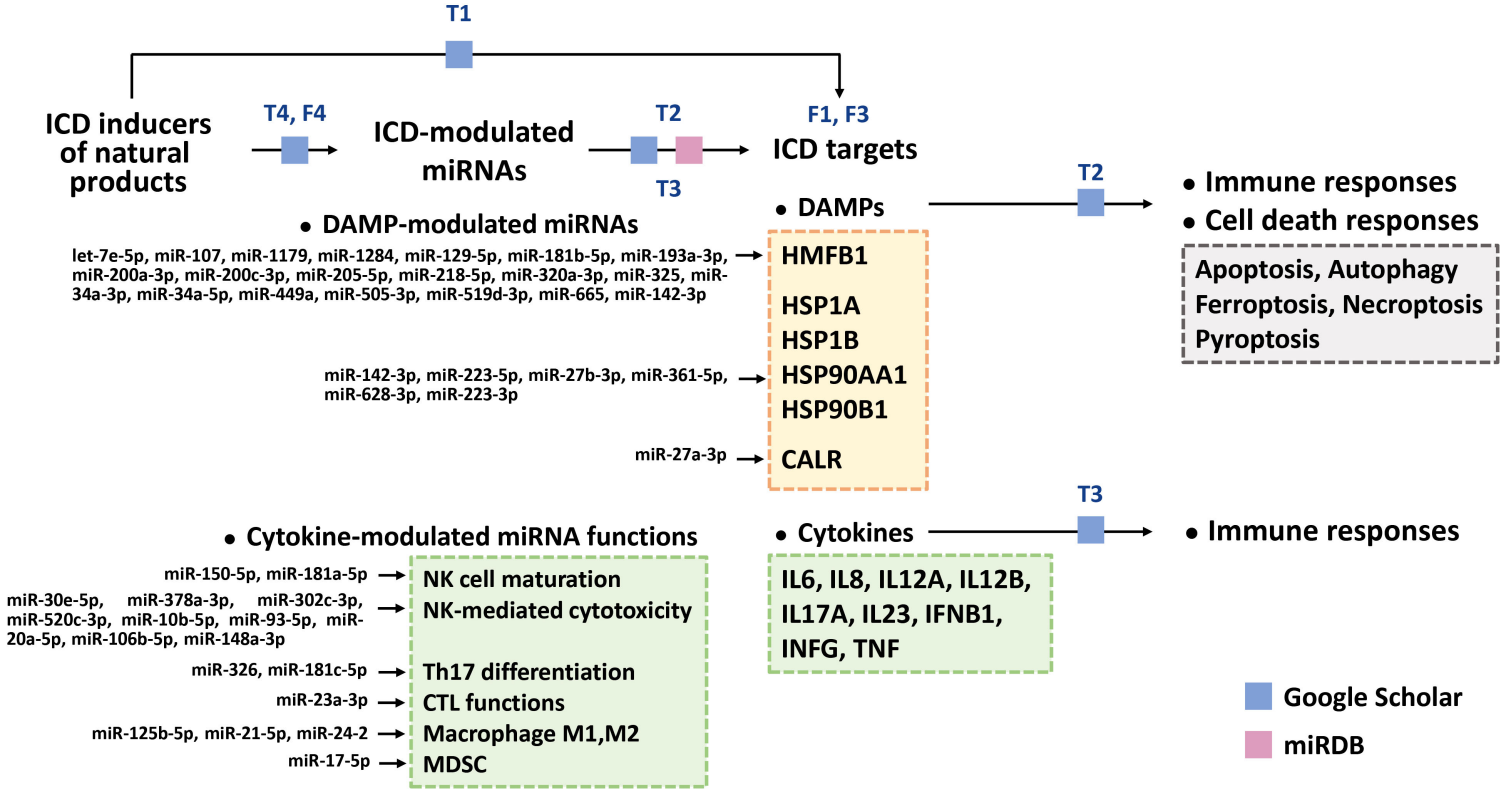
Arrangement of this review. Using Google Scholar, we organized natural products with ICD-inducing functions and their ICD targets, such as DAMPs and cytokines [**Table 1** (T1)]. ICD-modulating miRNAs that regulate DAMPs and cytokines were also retrieved [**Tables 2** and **3** (T2, T3)]. Based on our searches in Google Scholar and miRDB (270), the potential ICD targets (DAMPs (HMGB1, CALR, HSP1A, HSP1B, HSP90AA1, and HSP90B1) [**Figure 3** (F3)] and cytokines [IL6, IL8, IL12A, IL12B, IL17A, IL23, IFNB1, INFG, and TNF)] for these miRNAs were collected (**Tables 2**, **3**). Moreover, the DAMP-modulated miRNAs (**Table 1**) targeting DAMPs are shown. In comparison, the functions of cytokine-modulated miRNAs (**Table 2**) are presented. For DAMP-modulating miRNAs, the immune and cell death responses were categorized (**Table 2**). Cell death responses, such as apoptosis, autophagy, ferroptosis, necroptosis, and pyroptosis, were also retrieved. For cytokine-modulating miRNAs, their immune responses and expression levels in cancer cells were categorized [**Table 3** (T3)]. Finally, we ascertained the relationship between ICD inducers of natural products and ICD-modulating miRNAs [**Table 4** (T4) and **Figure 4** (F4)].

This review illustrates the systemic connection between ICD (DAMP and cytokine responses), cell death responses (apoptosis, autophagy, ferroptosis, necroptosis, and pyroptosis), miRNAs, and natural products (**Figure 2**). With the help of bioinformatics, the potential ICD targets of ICD-modulating miRNAs in natural product studies were retrieved from miRDB (270). The detailed reports on ICD, natural products, and miRNAs from the literature were retrieved using Google Scholar. Finally, we propose the rationale that the ICD inducers among natural products modulate ICD targets that involve DAMPs and cytokines (Section 2). In this review, miRNAs that target DAMPs and cytokines were collected, and the immune and cell death responses were integrated (Sections 3 and 4). Moreover, the interaction between ICD-inducing natural products and ICD-modulating miRNAs is also explored (Section 5).

## 2 ICD-inducing natural products that modulate DAMPs and cytokines

**Figure 3:**
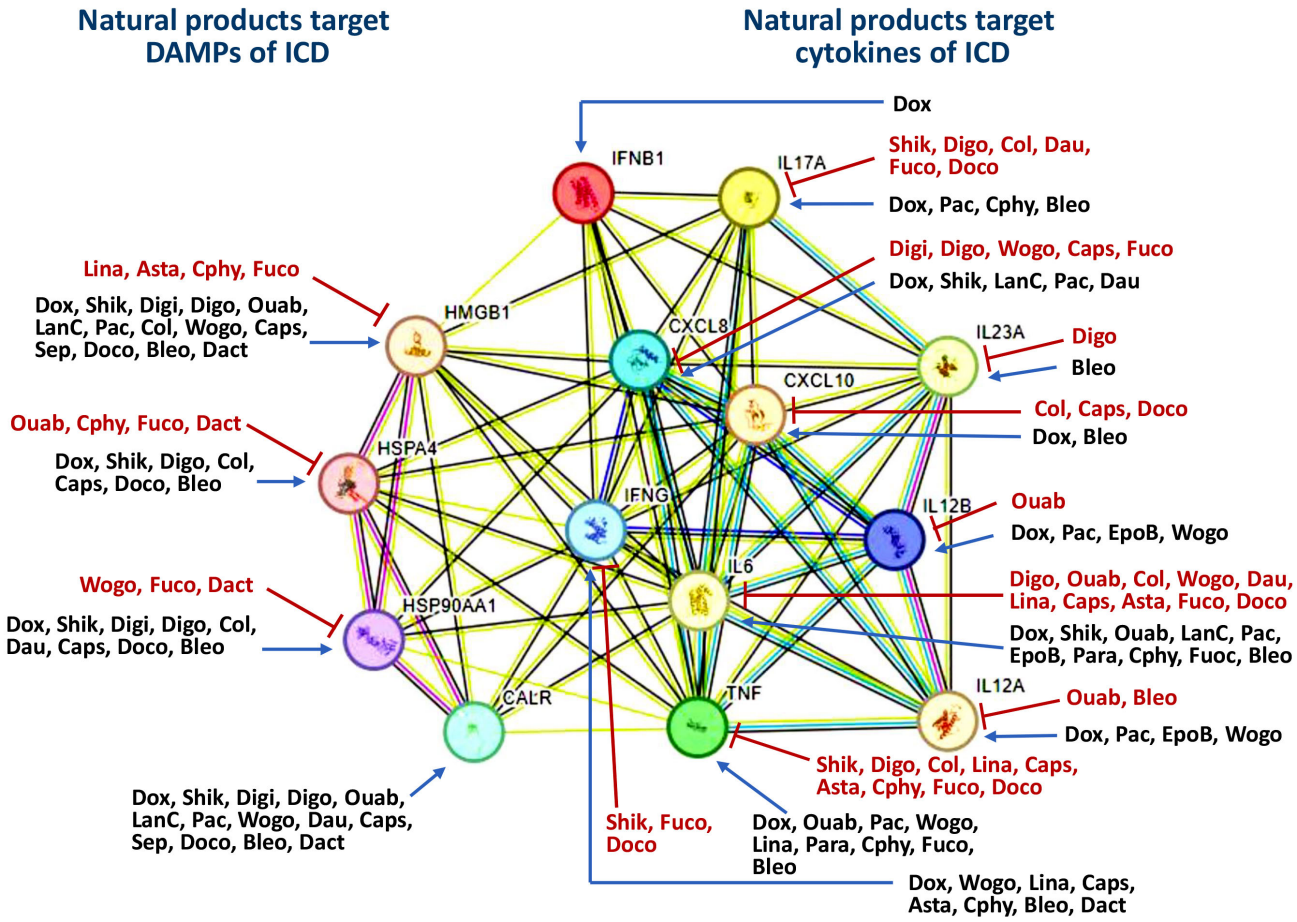
Classification of ICD inducers of natural products into different classes based on their targeting. The potential interaction for targets was analyzed by STRING database. All the information was derived from **Table 1**. The symbols of “T” and “arrow-line” indicate that natural products downregulate and upregulate their targets. HSP70 and HSP90 are labeled with HSPA4 and HSP90AA1 in the network, respectively. Dox, Doxorubicin; Shik, Shikonin; Digi, Digitoxin; Digo, Digoxin; Ouab, Ouabain; LanC, Lanatoside C; Pac, Paclitaxel; Col, Colchicine; EpoB, Epothilone B; Wogo, Wogonin; Dau, Daunorubicin; Lina, Linalool; Caps, Capsaicin; Asta, Astaxanthin; Para, Paramylon; Cphy, C-phycocyanin; Fuco, Fucoidan; Sep, Septacidin; Doco, Docosahexaenoic acid; Bleo, Bleomycin; Dact, Dactinomycin.

Many natural compounds can induce ICD (31, 32). Most natural product studies focus on cancer cells’ antiproliferative and immunomodulatory activity, while evidence for the induction of ICD in the immune system is limited. Consequently, we summarized the ICD-inducing natural products, focusing on the ICD-modulated responses to DAMPs and cytokines, particularly in cancer cells (**Table 1**). The ICD inducers of natural products are classified based on their targeting network, which is constructed by analyzing the STRING database (271) (**Figure 3**). The natural products are classified into five functions: cardiac glycosides, topoisomerase II inhibitors, anti-mitotic agents, antibiotics, and multiple functions (**Table 1**) (Sections 2.1-2.5).

**Table 1:** DAMP and cytokine responses of ICD-inducing natural products.

ICD inducers of natural products	DAMPs of ICD*				Cytokines of ICD*									
	HMGB1	CALR	HSP70	HSP90	CXCL8	CXCL10	IL6	IL12A	IL12B	IL23A	IL17A	TNF	IFNG	IFNB1
Cardiac glycosides														
Digitoxin (33)	(33)	(33)		(33)	(34) ↓									
Digoxin (33)	(35)	(35)	(36)	(33)	(34, 37) ↓		(37) ↓			(38) ↓	(39) ↓	(37) ↓		
Ouabain (40)	(40)	(40)	(41) ↓				(42), (43) ↓	(44) ↓	(44) ↓		(45) ↓	(42)		
Lanatoside C (40)	(40)	(40)			(46)		(46)							
Topoisomerase II inhibitors														
Doxorubicin (1, 47)	(14)	(14)	(14)	(14)	(48)	(49)	(50)	(51)	(51)		(52)	(48)	(49, 51)	(49)
Daunorubicin (53)		(53)	(53)	(53)			(54) ↓				(55) ↓			
Dactinomycin (56)	(56)	(56)	(57) ↓	(57) ↓									(56)	
Anti-mitotic agents														
Paclitaxel (58)	(58)	(59)			(60)	(61)	(60)	(62)	(62)		(63)	(64)		
Colchicine (65)	(66)		(66)	(66)		(67) ↓	(68) ↓				(69) ↓	(70) ↓		
Epothilone B (71)							(71)	(71)	(71)					
Antibiotics														
Septacidin (72)	(72)	(72)												
Bleomycin (73)	(74)	(74)	(75)	(76)		(77)	(78)	(79) ↓		(80)	(80)	(78)	(74)	
Multiple functions														
Shikonin (81)	(81)	(82)	(81)	(81)	(83)		(83)				(84) ↓	(85) ↓	(84) ↓	
Wogonin (86)	(86)	(86)		(87) ↓	(88) ↓		(88) ↓	(89)	(89)			(89)	(90)	
Linalool (91)	(92) ↓						(92) ↓					(91), (92) ↓	(91)	
Capsaicin (94)	(94)	(94)	(94)	(94)	(95) ↓	(96) ↓	(95, 96) ↓					(95, 96) ↓	(97)	
Astaxanthin (98)	(99) ↓						(100, 101) ↓					(102) ↓	(98)	
Paramylon (103)							(103)					(103)		
C-phycocyanin (104)	(105) ↓		(106) ↓				(104)				(107)	(104), (105) ↓	(107)	
Fucoidan (108)	(109) ↓		(110) ↓	(110) ↓	(111) ↓		(108), (109, 111) ↓				(275) ↓	(108), (109, 111) ↓	(275) ↓	
Docosahexaenoic acid (112)	(112)	(112)	(113)	(112)		(114) ↓	(115) ↓				(116) ↓	(115) ↓	(117) ↓	

* ↓ indicates that some natural products downregulate the DAMPs and cytokines of ICD. Except for those indicated with the symbol ↓, the natural products exhibit inducing effects on these targets without a symbol.

### 2.1 Cardiac glycosides

#### 2.1.1 Digitoxin and digoxin

Digitoxin (33) and digoxin (35), foxglove (*Digitalis purpurea*)-derived cardenolides, upregulate ICD-related molecules such as DAMPs (HSP90, CALR, and HMGB1) in osteosarcoma cells (**Table 1**) (**Figure 3**). Digoxin also upregulates HSP70 levels in patients with chronic heart failure (36).

These natural products also induce ICD-modulated cytokine responses (**Table 1**) (**Figure 3**). Digitoxin downregulates CXCL8 in cultured lung epithelial cells (34). Similarly, digoxin downregulates various ICD-modulating cytokines, as well as CXCL8, IL6, and TNF, in peripheral blood mononuclear cells (37). Digoxin downregulates bortezomib-induced IL23A in brain vascular endothelial cells (38) and IL17A in colonic mucosa (39). Accordingly, digitoxin and digoxin regulate DAMPs’ and ICD-modulated cytokines’ functions.

#### 2.1.2 Ouabain and lanatoside C

Ouabain and lanatoside C, the cardiac glycosides, induce DAMP responses (CALR exposure, ATP secretion, and HMGB1 release) (**Table 1**) (**Figure 3**) (40). Moreover, these natural products regulate ICD-modulating cytokines. For example, ouabain upregulates IL6 and TNF in peripheral blood mononuclear cells (42). In comparison, it downregulates IL6 signaling (43), TNF-induced IL12 (44), and IL17A (45) in cultured skeletal muscle cells, dendritic cells, and bronchial epithelial cells, respectively. Additionally, lanatoside C upregulates IL6 and CXCL8 in pericytes (46). Accordingly, ouabain and lanatoside C regulate DAMPs and ICD-modulated cytokines’ functions.

### 2.2 Topoisomerase II inhibitors

#### 2.2.1 Doxorubicin

Doxorubicin upregulates DAMPs (CARL, HSP70, and HSP90), causing them to translocate to the cell surface and causing the release of HMGB1 in leukemia, ovarian, and prostate cancer cells (**Table 1**) (**Figure 3**) (14). In terms of ICD-modulating cytokines, doxorubicin upregulates the production of IL6 (50) and IFNG (272). IL17A enhances the doxorubicin sensitivity of breast cancer cells (52). Moreover, doxorubicin upregulates CXCL8 and TNF in lung cancer cells (48) and IL12-induced IFNG in xenografted breast cancer (51). Accordingly, it regulates DAMPs and ICD-modulating cytokines’ functions.

#### 2.2.2 Daunorubicin

Daunorubicin, a *Streptomyces peucetius*-derived antibiotic, induces DAMPs (CARL exposure and the release of HSP70/HSP90) in acute myeloid leukemia cells (53) (**Table 1**) (**Figure 3**). It also modulates several ICD-modulating cytokines. Histone deacetylase 8 (HDAC8) is upregulated in daunorubicin-resistant AML cells. In contrast, the inhibition of HDAC8 promotes daunorubicin sensitivity by downregulating IL6 (54). Moreover, the upregulation of AKT and IL17A enhances the daunorubicin resistance of B cell acute lymphoblastic leukemia (ALL) cells (55), suggesting that daunorubicin downregulates IL17A.

#### 2.2.3 Dactinomycin

Actinomycin D (dactinomycin), a natural chromopeptide, promotes the release of HMGB1 and IFNG and the exposure of CALR in osteosarcoma cells (**Table 1**) (**Figure 3**) (56). In chicken embryo cells, TGFβ upregulates HSPA4 and HSP90AA1, which are downregulated by dactinomycin (57).

Accordingly, bleomycin and dactinomycin modulate DAMPs and ICD-modulated cytokines’ effects.

### 2.3 Microtubule inhibitors

#### 2.3.1 Paclitaxel

Paclitaxel is an ICD inducer whose activity is characterized by the release of HMGB1 in osteosarcoma cells (58) and upregulation of CALR in lung cancer cells (**Table 1**) (**Figure 3**) (59). Moreover, paclitaxel regulates ICD-modulating cytokines. For example, it upregulates CXCL8 and IL6 in ovarian cancer patients (60) and CXCL10 expression in lung cancer cells (61). In *in vivo* studies, paclitaxel upregulated the expression of IL12 in macrophages in fibrosarcoma-xenografted mice (62), of IL17A in solid Ehrlich carcinoma mouse models (63), and of TNF in hippocampus tissue (64).

#### 2.3.2 Colchicine and epothilone B

Originally isolated from *Colchicum autumnale*, colchicine triggers dendritic cell maturation (65) and upregulates or downregulates DAMPs or ICD-modulating cytokines (**Table 1**) (**Figure 3**). It upregulates DAMPs (HMGB1, HSPA4, and HSP90AA1) in melanoma cells, for example (66). In comparison, colchicine downregulates CXCL10 (67). Moreover, it downregulates IL6 (68), IL17A (69), and LPS-induced TNF (70) expression in cardiac fibroblasts, atrial fibrillation patients, and macrophages, respectively. Additionally, epothilone B (patupilone), a *Sorangium cellulosum*-derived microtubular inhibitor, is an ICD inducer in ovarian cancer cells that upregulates IL12 and IL6 (71).

Accordingly, paclitaxel, colchicine, and epothilone B modulate DAMPs and ICD-modulated cytokines’ functions.

### 2.4 Antibiotics

Septacidin, an L-heptopyranose isolated from *Streptomyces fibriatus*, enhances CALR exposure and ATP and HGMB1 secretion from osteosarcoma cells (**Table 1**) (**Figure 3**) (72). Bleomycin, a *Streptomyces verticillus*-derived antibiotic, induces HMGB1, CALR, and IFNG expression in colon cancer cells (73). It upregulates HSPA4 in the lung epithelium (75) and HSP90AA1 in the interstitial lung fibroblasts (76) of mice. Moreover, bleomycin regulates several ICD-modulating cytokines. For example, it increases γδ T-cell populations and upregulates CXCL10, affecting inflammation (77). Bleomycin upregulates IL6 and TNF in the lung homogenates of CBA/J mice (78), as well as IL17A and IL23A in C57BL/6 mice (80). In comparison, the transcription factor Fli-1 is downregulated in systemic sclerosis. Bleomycin downregulates IL12A in Fli-1^+/−^ mice, a skin fibrosis model (79).

### 2.5 Multiple functions

#### 2.5.1 Shikonin and wogonin

Shikonin promotes the release of HSP70, HSP90, and HMGB1 in melanoma cells by enhancing immunogenic apoptosis (**Table 1**) (**Figure 3**) (81). It also upregulates DAMPs (HMGB1, HSP70, and CALR) in glioma cells (82). In comparison, it inhibits several ICD-modulating cytokines. For example, it suppresses TNF-induced IL6 and CXCL8 production in human periodontal ligament cells (273) and inhibits T cell proliferation by downregulating IFNG and IL17A (84). It also downregulates TNFA in rheumatoid arthritis-like cell models (85).

Wogonin, a *Scutellaria baicalensis*-derived natural product, induces DAMP responses (**Table 1**) (**Figure 3**). For example, it promotes ICD and ER stress in dendritic cells, causes CALR exposure on the cell surface, and triggers the release of HMGB1 and ATP (86). Subsequently, these released molecules cause dendritic cells to secrete cytokines (86). In breast cancer cells, wogonin inhibits proteins downstream of HSP90AA1 such as EGFR, Cdk4, and survivin (87).

Moreover, wogonin regulates ICD-modulating cytokines. For example, it suppresses IL-1β’s promotion of IL6 and CXCL8 expression in retinal pigment epithelial cells (88). It also induces IL12 and TNF expression in breast cancer cells (89) and downregulates IFNG generation in splenocytes (90).

Accordingly, shikonin and wogonin regulate DAMPs and ICD-modulated cytokines’ functions.

#### 2.5.2 Linalool and capsaicin

Linalool improves Th1 cellular immunity in breast cancer cells by inducing the release of IFNG and TNF in lymphocytes (**Table 1**) (**Figure 3**) (91). It downregulates HMBG1, TNF, and IL6 in cisplatin-induced acute kidney injury in rat models (92).

Capsaicin, a red pepper-derived compound, suppresses the proliferation of many cancer cells (**Table 1**) (**Figure 3**) (93). It induces DAMPs in human bladder cancer cells by upregulating HMBG1, CALR, HSPA4, and HSP90AA1 (94). Regarding ICD-modulating cytokines, capsaicin downregulates TNF, IL6, and CXCL8 in monocytes (95). It has an anti-inflammatory effect in wound healing by downregulating TNF, IL6, and CXCL10 (96). In comparison, it upregulates IFNG in murine Peyer’s patch cells (97).

Accordingly, daunorubicin, linalool, and capsaicin regulate DAMPs and ICD-modulated cytokines’ functions.

#### 2.5.3 Astaxanthin, paramylon, and C-phycocyanin

Astaxanthin, a carotenoid derived from the green alga *Hematococcus pluvialis*, induces ICD (**Table 1**) (**Figure 3**) (1). It attenuates spinal cord edema by downregulating HMGB1 in rat models (99). Moreover, it modulates several ICD-modulating cytokines, such as by enhancing immunity through inducing the release of IFNG in lymphocytes (98). Astaxanthin downregulates IL6 expression in activated microglia (100) and cerulein-/resistin-stimulated pancreatic acinar cells (101) and TNF expression in LPS-treated macrophages (102).

Paramylon, a *Euglena gracilis*-derived beta-(1–>3)-D-glucan, induces ICD (**Table 1**) (**Figure 3**) (274). Regarding cytokine regulation, paramylon nanofibers upregulate TNF and IL6 mRNA expression in lymphocytes (103).

*Spirulina* microalgae-derived C-phycocyanin induces ICD (104). It enhances the secretion of IL6 and TNF in murine macrophages (**Table 1**) (**Figure 3**) (104). HMGB1 induces ulcers, which, in turn, are suppressed by its downregulation. C-phycocyanin attenuates ethanol-induced gastric ulcers in rats by downregulating HMGB1 and TNF (105). Similarly, dietary C-phycocyanin increases the lifespan of *Drosophila melanogaster* by downregulating HSPA4, a member of the HSP70 family (106). In comparison, some cytokines are induced by C-phycocyanin, which upregulates IFNG and IL17A in BALB/c mice (107).

Accordingly, astaxanthin, paramylon, and C-phycocyanin can regulate DAMPs and ICD-modulated cytokines.

#### 2.5.4 Fucoidan and docosahexaenoic acid

Fucoidan can modulate DAMPs and ICD-modulated cytokines (**Table 1**) (**Figure 3**). For example, it inhibits HSPA4 and HSP90AA1 protein expression in liver cancer cells (110), while downregulating HMGB1, IL6, and TNF levels in ischemia–reperfusion rats (109). It also induces IL6 and TNF expression, promoting the maturation of spleen dendritic cells (108). Fucoidan-rich *Ascophyllum nodosum* extract (Ascophyscient^®^) downregulates TNF, IL6, and CXCL8 in bronchial epithelial cells (111). Fucoidan downregulates IL17A and IFNG in T helper cells (Th1/Th2/Th17) (275).

Docosahexaenoic acid (DHA) enhances myeloma apoptosis by improving CALR exposure, HMGB1, and HSP90 secretion and activating dendritic cells (**Table 1**) (**Figure 3**) (112). DHA also upregulates HSPA4 in rainbow trout leukocytes (113). In comparison, it downregulates several ICD-modulating cytokines, such as Cxcl10 in the lupus flaring mouse model (114). DHA downregulates IL6 and TNF secretion in LPS-activated dendritic cells, preventing their maturation (115). It also downregulates IL17A in T cells in psoriatic skin models (116). Moreover, DHA intake downregulates IFNG production in mice (117).

Accordingly, fucoidan, septacidin, and DHA show a regulating effect on DAMP and ICD-modulated cytokines.

## 3 DAMP-modulating miRNAs

Several miRNAs that modulate DAMPs can regulate ICD and tumor proliferation (2). miRNAs exhibit tumor-promoting and tumor-suppressing effects by targeting various DAMPs, whose expression levels are changed in cancers (2). Therefore, the ICD response of the immune system and the cell death responses of ICD-modulating miRNAs need further investigation.

This section presents an overview of the impacts of miRNAs on immune and cell death responses. The immune responses affected by DAMP-modulating miRNAs (Section 3.1), and cell death responses affected by DAMP-modulating miRNAs (Section 3.2) and DAMP-targeting miRNAs (Section 3.3) are described (**Table 2**).

**Table 2:** Potential targets and immune and cell death responses for DAMP-modulating miRNAs.

ICD-modulating miRNAs	Targets for		Responses for					
	DAMP	miRDB *^1^	Immune	Cell death *^2^				
				Apoptosis	Autophagy	Ferroptosis	Necroptosis	Pyroptosis
let-7e-5p	HMGB1 (118)	IL6		chondrocytes ↓ (119)	chondrocytes (119)			
miR-107	HMGB1 (120)			glioma (121)	breast ca (120)			chondrocytes ↓ (122)
miR-1179	HMGB1 (123)			gastric ca (123)	oral ca ↓ (124)			
miR-1284	HMGB1 (125)	IL12B		cervical ca (125)				
miR-129-5p	HMGB1 (126)			gastric ca (126)	colon ca ↓ (127)	intestinal epithelial cells ↓ (128)		pheochromocytoma (129)
miR-181b-5p	HMGB1 (130)	CALR, TNF, HSP90B1	B, T cells (119)	AML (130)	gallbladder ca (132)	chondrocytes (133)	lymphocytes ↓ (134)	endothelial cells ↓ (135)
miR-193a-3p	HMGB1 (136)			colon ca (137)	liver ca ↓ (138)	cardiomyocytes ↓ (139)		
miR-200a-3p	HMGB1 (140)	IL17A		prostate ca (141)	cardiomyocyte (142)	myocardial cells ↓ (143)	intestinal epithelial cells ↓ (144)	aortic endothelial cells ↓ (145)
miR-200c-3p	HMGB1 (146)		Macrophage (147)	trabecular meshwork cells ↓ (148)	prostate ca (149)			retinal vascular cells (150)
miR-205-5p	HMGB1 (151)			gastric ca (152)	prostate ca ↓ (153)	MI/R injury ↓ (154)		
miR-218-5p	HMGB1 (155)			cervical ca (156), RASFs (157)	RASFs (157)		glioblastomas (158)	
miR-320a-3p	HMGB1 (159)	HSPA4, IL12B		B cells ↓ (160)	B cells ↓ (160)	breast ca (161)		
miR-325	HMGB1 (162)			gastric ca (163)	cardiomyocytes (164)			
miR-34a-3p	HMGB1 (165)	TNF		retinoblastoma (165)	retinoblastoma (165)		meningioma cells (166)	liver ca (167)
miR-34a-5p	HMGB1 (168)	HSPA1B, CXCL10	CD8+ T cells (169)	lung ca (170)	ovarian granulosa cells (171)			cardiomyocytes (172)
miR-449a	HMGB1 (173)	HSPA1B, CXCL10		liver ca (174)	T cells ↓ (175)			
miR-505-3p	HMGB1 (176)	IFNG		lung ca (177)	neuron ↓ (178)			
miR-519d-3p	HMGB1 (179)			liver ca (180)	liver ca (180)			
miR-665	HMGB1 (181)			lung ca ↓ (182), breast ca ↓ (183)	macrophages ↓ (184)			MIRI (185)
miR-142-3p	HMGB1 (186), HSPA1B (187)		CD3+ spleen lymphocytes (188)	breast ca (186)	breast ca ↓ (186)	liver ca (189)		myocardial injury ↓ (190)
miR-223-5p	HSPA1A (191)			spinal cord injury rats (192)	prostate ca (193)		ischemic/ reperfused hearts ↓ (194)	
miR-27b-3p	HSP90AA1 (195)		Th1, Th2, Th17, Treg (196)	breast ca (197)				
miR-361-5p	HSP90AA1 (198)			ovarian ca ↓ (199)	gastric ca ↓ (200)			
miR-628-3p	HSP90AA1 (201)			lung ca (201)				
miR-223-3p	HSP90B1 (202)		M2 macrophages (203)	osteosarcoma (202)	liver ca ↓(204)	podocytes ↓ (205)	macrophage ↓ (206)	cardiomyocyte (207)
miR-27a-3p	CALR (2)			colon ca ↓ (208), pre-osteoblast cells ↓ (209)	pre-osteoblast cells (209)	lung ca ↓ (210)		

*^1^. Target candidates include DAMP- and cytokine-modulating genes, as **Figure 2** (section 1.2) shows. *^2^. ↓ indicates that miRNAs downregulate the cell death responses (right). Except for those indicated with the symbol ↓, the miRNAs exhibit inducing effects on these cell death responses without a symbol. ca, cancer cells; Th, helper T cells; Treg, regulatory T cells; RASFs, rheumatoid arthritis synovial fibroblasts; MIRI, myocardial ischemia–reperfusion injury.

### 3.1 Immune responses affected by DAMP-modulating miRNAs

Some DAMP-targeting miRNAs modulate immune responses (**Table 2**). For example, miR-181b-5p upregulation in chronic lymphocytic leukemia B cells enhances cytotoxic T cell function, inhibiting tumor growth (131). miR-200c-3p inhibits the tumor-infiltrating function of macrophages (147), while miR-34a-5p enhances that of CD8+ T lymphocytes (169).

miR-142-3p is highly expressed in immune cells, such as CD3+ spleen lymphocytes derived from experimental autoimmune encephalomyelitis mice, compared to Complete Freund’s Adjuvant CD3+ (188) (**Table 2**). miR-223-3p enhances the differentiation of macrophages via M2 polarization (203).

miR-27b-3p enhances ammonia-triggered apoptosis by targeting TNF receptor-associated death domain (TRADD), Fas-associated death domain (FADD), and apoptotic protease activating factor-1 (APAF1) (**Table 2**). Ammonia induces apoptosis and immunosuppression by causing a T helper cell type 1 (Th1)/Th2 imbalance and regulatory T cell (Treg)/Th17 imbalance in chicken peripheral blood lymphocytes (196). Accordingly, miR-27b-3p promotes immunosuppression through Th1/Th2 and Treg/Th17 imbalances.

Notably, most of the miRNAs listed in **Table 2** are rarely investigated in terms of their involvement in the immune response. In the future, the immune-regulating function of these ICD-modulating miRNAs should be carefully and more thoroughly investigated.

### 3.2 Cell death responses of DAMP-modulating miRNAs

ICD is a general term that includes various cell death responses, such as apoptosis, autophagy, ferroptosis, pyroptosis, and necroptosis (276, 277). These responses exert synergistic effects by enhancing or suppressing antitumor immune responses (277). Currently, the cell death responses of ICD-modulating miRNAs are rarely highlighted. To address this gap, we outline miRNAs that modulate ICD (**Table 2**) by regulating apoptosis, autophagy, ferroptosis, necroptosis, and pyroptosis in the following (Sections 3.3 to 3.5).

### 3.3 DAMP-targeting miRNAs

DAMPs, such as HMGB1, HSP70, HSP90, and CALR (Sections 3.3.1-3.3.4), are targeted by various miRNAs (**Table 2**). Based on a search of miRDB, we describe the potential targets of DAMPs and cytokines (270). The cell death responses they influence, including apoptosis, autophagy, ferroptosis, necroptosis, and pyroptosis, are summarized (**Table 2**) (**Figure 2**).

#### 3.3.1 HMGB1-targeting miRNAs

A series of investigations have reported that many miRNAs have HMGB1-modulating functions. By targeting HMGB1, these miRNAs (let-7e-5p, miR-107, miR-1179, miR-1284, miR-129-5p, miR-181b-5p, miR-193a-3p, miR-200a-3p, miR-200c-3p, miR-205-5p, miR-218-5p, miR-320a-3p, miR-325, miR-34a-3p, miR-34a-5p, miR-449a, miR-505-3p, miR-519d-3p, miR-665, and miR-142-3p) exhibit anticancer effects associated with various cell death responses (**Table 2**) (**Figure 2**) (Sections 3.3.3.1-3.3.3.6).

##### 3.3.1.1 let-7e-5p, miR-107, miR-1179, and miR-1284

let-7e-5p overexpression suppresses the proliferation and migration of thyroid cancer cells by targeting and downregulating HMGB1 (118) (**Table 2**). Regarding cell death responses, let-7e-5p downregulation triggers apoptosis but blocks autophagy in articular chondrocytes (119). miR-107 is underexpressed in breast cancer cell lines and tissues. Its overexpression inhibits proliferation, migration, and autophagy by downregulating HMGB1 (120), while its upregulation triggers apoptosis in glioma cells (121). Moreover, miR-107 enhances proliferation by downregulating the LPS-triggered pyroptosis of chondrocytes (122) (**Table 2**).

Gastric cancer shows low levels of miR-1179. The overexpression of miR-1179 inhibits gastric cell migration and proliferation and promotes apoptosis by targeting HMGB1 (123) (**Table 2**), while its downregulation promotes autophagy in oral cancer cells (124). Moreover, miR-1284 is underexpressed in cervical cancer cell lines and tissues. Its overexpression sensitizes cells to cisplatin and promotes apoptosis in cervical cancer cells by downregulating HMGB1 (125) (**Table 2**).

Accordingly, let-7e-5p, miR-107, miR-1179, and miR-1284 modulate cell death responses.

##### 3.3.1.2 miR-129-5p, miR-181b-5p, and miR-193a-3p

miR-129-5p is less expressed and HMGB1 is more expressed in gastric cancer than in normal tissues (126) (**Table 2**). The former regulates several cell death responses. For example, its upregulation triggers apoptosis in gastric cancer cells by downregulating HMGB1. miR-129-5p improves the radiosensitization of colon cancer cells by downregulating autophagy (127). It blocks ferroptosis in intestinal epithelial cells (128), while a miR-129-5p antagomir attenuates LPS-triggered neuronal pyroptosis in rat pheochromocytoma cells (129) (**Table 2**).

miR-181b-5p is underexpressed in AML patients. Its overexpression sensitizes cells to doxorubicin, suppressing proliferation and inducing apoptosis in AML cells, by targeting HMGB1 (130) (**Table 2**). This miRNA also regulates several cell death responses. For example, it inhibits ginsenoside Rg3’s suppression of proliferation in gallbladder cancer cells by upregulating autophagy (132). miR-181b-5p is highly expressed in osteoarthritic cell models, while in osteoarthritic chondrocytes, its downregulation inhibits ferroptosis by upregulating GPX4 (133). Atrazine triggers inflammation and necroptosis in carp lymphocytes by downregulating miR-181-5p (134). The overexpression of the latter attenuates NLRP3 inflammasome-mediated pyroptosis in vascular endothelial cells (135) (**Table 2**).

miR-193a-3p is more downregulated in lung cancer than in normal cells (278). It inhibits the migration and proliferation of lung cancer cells and downregulates HMGB1 (136) (**Table 2**). miR-193a-3p modulates several cell death responses. For example, it suppresses proliferation and causes apoptosis in colon cancer cells (137). miR-193a-3p mimics downregulated autophagy in liver cancer cells, which was reverted by miR-193a-3p inhibitors (138). miR-193a-3p downregulation caused congenital heart disease by upregulating ferroptosis in rat cardiomyocytes (139) (**Table 2**).

Accordingly, miR-129-5p, miR-181b-5p, and miR-193a-3p modulate cell death responses.

##### 3.3.1.3 miR-200a-3p, miR-200c-3p, and miR-205-5p

miR-200a-3p shows low expression in liver cancer, while HMGB1 is highly expressed (140) (**Table 2**). This miRNA inhibits liver cancer cell proliferation by downregulating HMGB1 and modulates several cell death responses. For example, miR-200a-3p upregulation suppresses proliferation and promotes apoptosis (141) in prostate cancer cells. Its upregulation also attenuates diabetic cardiomyopathy damage in mice by upregulating autophagy (142), while its downregulation suppresses hypoxia/reoxygenation (H/R)-triggered ferroptosis and protects the myocardial cells (143). Moreover, miR-200a-3p mimics suppress lipopolysaccharide-induced inflammation and necrosis in intestinal epithelial cells (144). miR-200a-3p’s downregulation suppresses pyroptosis in human aortic endothelial cells (145) (**Table 2**).

miR-200c-3p shows low expression in non-small cell lung cancer (NSCLC) (146). It suppresses epithelial–mesenchymal transition, invasion, and migration in lung cancer cells by downregulating HMGB1 (146) (**Table 2**). It modulates several cell death responses. For example, miR−200c−3p mimics promote proliferation and suppress apoptosis in trabecular meshwork cells (148). miR-200c-3p induces autophagy in prostate cancer cells (149), and its downregulation attenuates high glucose-induced pyroptosis in human retinal microvascular endothelial cells (HRMECs) (150), suggesting that miR-200c-3p has a pyroptosis-inducing function (**Table 2**).

miR-205-5p shows low expression in breast cancer cells, enhancing EMT and invasion (151). Its overexpression suppresses the migration and proliferation of breast cancer cells by targeting HMGB1 (**Table 2**). Similarly, miR-205-5p is underexpressed in gastric cancer. Its overexpression suppresses proliferation and metastasis and triggers apoptosis in gastric cancer cells (152). miR-205 inhibits autophagy, improving the cisplatin sensitivity of prostate cancer cells (153). lncAABR07025387.1, highly expressed in myocardial ischemia/reperfusion (MI/R) injury, sponges miR-205-5p and upregulates ferroptosis (154) (**Table 2**), suggesting that miR-205-5p downregulates ferroptosis.

Accordingly, miR-200a-3p, miR-200c-3p, and miR-205-5p modulate cell death responses.

##### 3.3.1.4 miR-218-5p, miR-320a-3p, and miR-325-3p

miR-218-5p is underexpressed in NSCLC (279). The overexpression of miR-218-5p inhibits migration in lung cancer cells by targeting and downregulating HMGB1 (155) (**Table 2**). miR-218-5p modulates several cell death responses. For example, its upregulation promotes apoptosis in cervical cancer cells (156). miR-218-5p silencing suppresses proliferation by triggering apoptosis and autophagy in rheumatoid arthritis synovial fibroblasts (RASFs) (157). Moreover, necrotic and hypoxic mesenchymal glioblastomas exhibit low miR-218-5p levels (158), suggesting that miR-218-5p downregulates necrosis in mesenchymal glioblastoma cells.

miR-320a-3p is downregulated in liver cancer tissues (159). Its overexpression suppresses the invasion and metastasis of liver cancer cells by targeting HMGB1 (**Table 2**). miR-320a-3p modulates several cell death responses. For example, LINC00963 overexpression induces the apoptosis and autophagy of diffuse large B-cell lymphoma by downregulating miR-320a, which is reversed by miR-320a-3p mimics (160). LINC00460 downregulation inhibits proliferation but enhances ferroptosis in breast cancer cells by upregulating miR-320a-3p (161) (**Table 2**).

In lung cancer patients, miR-325-3p and HMGB1 are underexpressed and overexpressed, respectively (162) (**Table 2**). The overexpression of miR-325-3p inhibits the proliferation of lung cancer cells by targeting HMGB1. miR-325-3p modulates several cell death responses. For example, it inhibits the proliferation of gastric cancer cells by triggering apoptosis (163). Its downregulation suppresses myocardial ischemia/reperfusion-induced autophagy and cell death in cardiomyocytes (164) (**Table 2**).

Accordingly, miR-218-5p, miR-320a-3p, and miR-325-3p modulate cell death responses.

##### 3.3.1.5 miR-34a-3p, miR-34a-5p, and miR-449a

miR-34a-3p is expressed at low levels in retinoblastoma cells (280). Its overexpression inhibits proliferation and autophagy and triggers apoptosis in retinoblastoma cells by targeting HMGB1 (165) (**Table 2**). It also modulates other cell death responses. For example, anti-miR-34a-3p inhibits late apoptosis and necrosis in meningioma cells (166). LncRNA SNHG7 suppresses pyroptosis in liver cancer cells by sponging miR-34a-3p (167). This suggests that miR-34a-3p promotes apoptosis, necrosis, and pyroptosis.

miR-34a-5p shows low expression in colon cancer tissues (168). Its upregulation in colon cancer cells inhibits proliferation and migration by targeting HMGB1 (**Table 2**). It modulates other cell death responses. For example, apigenin triggers apoptosis in lung cancer cells by upregulating miR-34a-5p (170). miR-34a-5p enhances autophagy in chicken ovarian granulosa cells (171), while its downregulation inhibits pyroptosis in doxorubicin-induced cardiomyopathy (172) (**Table 2**).

miR-449a is poorly expressed in lung cancer cells and tissues (173). Its upregulation suppresses the proliferation and migration of lung cancer cells by targeting HMGB1 (**Table 2**). miR-449a modulates several cell death responses, suppresses proliferation and promotes apoptosis in liver cancer cells (174), and suppresses autophagy in T lymphocytes (175) (**Table 2**).

Accordingly, miR-34a-3p, miR-34a-5p, and miR-449a modulate cell death responses.

##### 3.3.1.6 miR-505-3p, miR-519d-3p, miR-665, and miR-142-3p

miR-505-3p is downregulated in liver cancer cells. Its overexpression inhibits the proliferation and invasion of liver cancer cells by targeting HMGB1 (176) (**Table 2**). miR-505-3p modulates other cell death responses. For example, its upregulation promotes apoptosis in lung cancer cells, which is reversed by its downregulation (177). miR-505-3p blocks autophagy in rat primary neurons by downregulating HMGB1 (178) (**Table 2**).

miR-519d-3p shows low expression in lung cancer tissues, promoting proliferation and migration by upregulating HMGB1 (179) (**Table 2**). Hence, it potentially targets HMGB1. Moreover, it triggers apoptosis and autophagy in liver cancer cells (180).

miR-665 is underexpressed in glioma (181). Its upregulation inhibits glioma cell proliferation, migration, and invasion by downregulating HMGB1 (**Table 2**). miR-665 modulates several cell death responses. For example, its mimics stimulate cell proliferation and suppress lung (281) and breast (183) cancer cell apoptosis. lncRNA MIAT promotes autophagy in Bacillus Calmette-Guerin (BCG)-infected macrophages by sponging miR-665 (184), suggesting that the latter inhibits macrophage autophagy. Moreover, dexmedetomidine downregulates miR-665, attenuating myocardial ischemia–reperfusion injury (MIRI) by inhibiting pyroptosis (185) (**Table 2**), suggesting that miR-665 promotes pyroptosis.

HMGB1 is a target of miR-142-3p (186) in breast cancer cells (**Table 2**). miR-142-3p is expressed at lower levels in breast cancer than in normal cells (282). Moreover, doxorubicin-resistant breast cancer cells exhibit lower levels of miR-142-3p than parental cells. miR-142-3p modulates several cell death responses. For example, its overexpression induces apoptosis and inhibits breast cancer cell autophagy, attenuating doxorubicin resistance by downregulating HMGB1 (186). miR-142-3p triggers ferroptosis in M1 macrophages, improving liver cancer development (189), and its upregulation attenuates coronary microembolization (CME)-induced pyroptosis in myocardial injury (190) (**Table 2**).

Accordingly, miR-505-3p, miR-519d-3p, miR-665, and miR-142-3p modulate cell death responses by targeting HMGB1.

#### 3.3.2 HSP70 and HSP90-targeting miRNAs

Some members of the HSP70 family were chosen as DAMP targets in this review, including HSPA1B and HSPA1A (Section 3.3.2.1). Some members of the HSP90 family were also selected, including HSP90AA1 and HSP90B1 (Section 3.3.2.2). These are targeted by several ICD-modulating miRNAs (**Table 2**) (**Figure 2**), and these HSP70 and HSP90-targeting miRNAs exhibit anticancer effects that are associated with various cell death responses (**Table 2**) (**Figure 2**).

##### 3.3.2.1 HSP70-targeted miRNAs

HSPA1B and HSPA1A are targeted by miR-223-5p and miR-142-3p (**Table 2**). Osteosarcoma overexpresses HSP70, which is downregulated by miR-223-5p (191). The overexpression of the latter enhances cisplatin sensitivity in osteosarcoma, while the upregulation of miR-142-3p in pancreatic cancer cells inhibits proliferation by targeting HSPA4 (HSP70) (187).

Regarding cell death responses, the apoptosis- and necroptosis-modulating effects of miR-223-5p are summarized (**Table 2**). miR-223-5p inhibitors suppress apoptosis signaling, such as caspase-3 in spinal cord injury rats (192), suggesting that miR-223-5p induces apoptosis. Moreover, miR-223-5p and miR-223-3p jointly inhibit necroptosis in ischemic/reperfused mouse hearts (194).

##### 3.3.2.2 HSP90-targeted miRNAs

HSP90AA1 and HSP90B1 are targeted by miR-27b-3p/miR-361-5p/miR-628-3p and miR-223-3p, respectively (**Table 2**). miR-27b-3p is underexpressed in lung cancer (283). LncRNA KCNQ1OT1 enhances the proliferation of lung cancer cells by upregulating HSP90AA1 and downregulating miR-27b-3p (195). Accordingly, miR-27b-3p suppresses HSP90AA1 expression in lung cancer cells. Moreover, miR-27b-3p promotes tamoxifen-triggered apoptosis in breast cancer cells (197) (**Table 2**).

miR-361-5p shows lower levels in cervical cancer than in normal tissues and cells (198). Its upregulation suppresses EMT and the invasion of cervical cancer cells by targeting HSP90 (**Table 2**). miR-361-5p also triggers cell death responses. For example, ovarian cancer cells exhibit high levels of the miRNA, whereas, in these cells, its downregulation promotes apoptosis (199). Moreover, miR-361-5p inhibits autophagy’s ability to improve chemoresistance in gastric cancer cells (200). Sp1 knockdown inhibits prostate cell growth and hypoxia-triggered autophagy by upregulating miR-361-5p (193). In comparison, miR-628-3p mimics cause apoptosis and suppress the migration of lung cancer cells by targeting and downregulating HSP90AA1 (201) (**Table 2**).

By targeting HSP90B1, miR-223-3p can induce anticancer effects and cell death responses (**Table 2**). For example, osteosarcoma shows a low level of oncogenic heat shock protein 90 kDa beta member 1 (HSP90B1) (202), a member of the HSP90 family. The upregulation of miR-223-3p suppresses osteosarcoma cell proliferation and promotes apoptosis by downregulating HSP90B1.

miR-223-3p also triggers cell death responses (**Table 2**). For example, doxorubicin induces autophagy in liver cancer cells by downregulating miR-223-3p. The upregulation of the latter suppresses doxorubicin-triggered autophagy, improving the chemoresistance of liver cancer cells (204). miR-223-3p enhances pyroptosis in cardiomyocytes (207). In comparison, exosomal miR-223-3p isolated from mesenchymal stem cells inhibits HBV-X protein (HBx)-triggered ferroptosis in podocytes (205), as well as necroptosis in macrophage cell death (206) (**Table 2**).

#### 3.3.3 CALR-targeting miRNAs

miR-27a-3p targets CALR (2). It is overexpressed in colon cancer cells and tissues, improving proliferation, suppressing apoptosis (208) (**Table 2**) (**Figure 2**), and triggering pre-osteoblast cell autophagy (209). SLC7A11, which is highly expressed in lung cancer patients, triggers the ferroptosis of lung cancer cells by downregulating miR-27a-3p (210), suggesting that miR-27a-3p inhibits ferroptosis.

Following treatment with ICD inducers (oxaliplatin and mitoxantrone), miR-27a-3p knockdown induces more cell surface CALR and HMGB1 secretion by colon cancer cells compared to that observed in cells overexpressing miR-27a-3p (284).

#### 3.3.4 Potential targets for DAMPs and cytokines according to miRDB search

Based on a search of the miRDB, in addition to targeting HMGB1, miR-181b-5p, miR-320a-3p, and miR-34a-5p/miR-449a potentially target DAMPs (CALR/HSP90B1, HSPA4, and HSPA1B) (270) (**Table 2**) (**Figure 2**). let-7e-5p, miR-1284, miR-181b-5p, miR-200a-3p, miR-320a-3p, miR-34a-3p, miR-34a-5p, and miR-505-3p also potentially target ICD-modulating cytokines, such as IL6, IL12B, TNF, IL17A, IL12B, TNF, CXCL10, IFNG, and HSPA1B, which are co-targeted by HMGB1 according to a search of miRDB (270) (**Table 2**) (**Figure 2**).

## 4 Cytokine-modulating miRNAs

In addition to DAMPs, ICD inducers may promote the secretion of inflammatory cytokines such as IL6 and CXCL8 (IL8) from cancer cells. The DAMPs and cytokines activate dendritic cells and NK cells, which then release effector cytokines, stimulating CTL and Th1 cells to release IFNG and Th17 cells to release IL17 (15). Moreover, M0 macrophages are stimulated to differentiate into M1 or M2 macrophages by several cytokines. M1 macrophages regulate many inflammatory, cytotoxic, and tissue damage functions. In comparison, M2 macrophages inhibit inflammatory and immune functions and promote tissue repair (285). MDSCs are responsible for the immune suppression activity of macrophages and dendritic cells (11). Several miRNAs regulating the cytokines that modulate factors related to immune responses, such as NK cell maturation, NK-mediated cytotoxicity, Th17 differentiation, CTL function, macrophage M1/M2 polarization, and MDSC levels, have been summarized (**Table 3**) (**Figure 2**) (Sections 4.1-4.5).

**Table 3:** Potential miRDB targets, immune function, and expression in cancer cells for cytokine-modulating miRNAs.

Cytokine-modulating miRNAs	miRNA functions*	miRDB targets	Expression in cancer*
miR-150-5p	NK cell maturation ↑ (211)	HSP90B1, TNF	adult T cell leukemia (212)
miR-181a-5p	NK cell maturation ↑ (213)	HSP90B1, TNF, CALR	bladder (214)
miR-30e-5p	NK-mediated cytotoxicity (215)		breast (216), bladder (217)
miR-378a-3p	NK-mediated cytotoxicity (215)		gastric (218)
miR-302c-3p	NK-mediated cytotoxicity (219)		cervical (220)
miR-520c-3p	NK-mediated cytotoxicity (219)		lung (221)
miR-10b-5p	NK-mediated cytotoxicity (222)		breast (223)
miR-93-5p	NK-mediated cytotoxicity (224)		colon (225), ovary (226)
miR-20a-5p	NK-mediated cytotoxicity (227)		ovarian ↑ (227)
miR-106b-5p	NK-mediated cytotoxicity (224)		lung ↑ (228), colon (229)
miR-148a-3p	NK-mediated cytotoxicity ↑ (230)	HSP90B1	renal (230)
miR-326	Th17 differentiation ↑(231)		lung, gastric, breast (232)
miR-181c-5p	Th17 differentiation ↑(233)	HSP90B1, TNF, CALR	cervical (234), breast (235)
miR-23a-3p	CTL functions (236)	HSP90B1, IL12B	renal ↑ (237)
miR-125b-5p	Macrophage M1 ↑ (238)		breast (239), liver (240)
miR-21-5p	Macrophage M1 (241)	IL12A	oral ↑ (242)
miR-24-2	Macrophage M1, M2 ↑ (222)		renal ↑(222)
miR-17-5p	MDSC (243)		gastric ↑(244)

* ↑ indicates that some miRNAs upregulate their matched miRNA function and expression in cancer. Except for those indicated with the symbol ↑, the miRNAs exhibit inhibitory effects without a symbol. NK, natural killer cells; CTL, cytotoxic lymphocytes; MDSC, myeloid-derived suppressor cells.

### 4.1 NK cell maturation

NK cell maturation is enhanced by miR-150-5p and miR-181a-5p (**Table 3**) (**Figure 2**). By directly targeting c-Myb, miR-150-5p improves the development and maturation of NK cells (211). miR-181a-5p enhances NK cell maturation by downregulating nemo-like kinase (NLK), an inhibitor of Notch signaling (**Table 3**) (213). Moreover, miR-150-5p (212) and miR-181a-5p (214) are downregulated in adult T-cell leukemia (ATL) and bladder cancer cells. Accordingly, they both exhibit tumor-suppressive potential by improving NK cell maturation; however, this needs further validation.

### 4.2 NK-mediated cytotoxicity

Several miRNAs, such as miR-30e-5p, miR-378a-3p, miR-302c-3p, miR-520c-3p, miR-10b-5p, miR-20a-5p, miR-93-5p, and miR-106b-5p, have inhibitory effects on NK-mediated cytotoxicity, while miR-148a-3p has induces it (**Table 3**).

miR-30e-5p and miR-378a-3p inhibit NK cell cytotoxicity (215) (**Table 3**) (**Figure 2**). Moreover, these miRNAs also exhibit anticancer effects. Breast (216) and bladder (217) cancer tissues and cells show low levels of miR-30e-5p (**Table 3**). miR-378a-3p is underexpressed in gastric cancer. When it is upregulated, proliferation is inhibited and apoptosis is promoted in gastric cancer cells (218). Similarly, 1,25(OH)_2_D3 inhibits miR-302c-3p and miR-520c-3p expression, improving NK cell cytotoxicity in breast cancer cells (219), suggesting that miR-302c-3p and miR-520c-3p downregulate NK cell cytotoxicity. Moreover, miR-302c-3p (220) and miR-520c-3p (221) are downregulated in cervical and lung cancer cells. The overexpression of the former suppresses proliferation and induces apoptosis in cervical cancer cells.

MHC class I chain-related protein B (MICB) is a stress-induced ligand of the activating NK-cell receptor NKG2D. miR-10b-5p inhibits MICB expression, suppressing NK cell cytotoxicity and thereby promoting cancer immune evasion, suggesting that miR-10b-5p downregulates NK cell cytotoxicity (286) (**Table 3**). In comparison, and based on data from the TCGA database, breast cancer tissues express lower levels of miR-10b-5p than normal controls (223). Moreover, the overexpression of miR-93-5p and miR-106b-5p downregulates MICA expression (224), suppressing NK cell cytotoxicity (287). Colon (225) and ovarian (226) cancer tissues exhibit low levels of miR-93-5p (**Table 3**), the upregulation of which inhibits the proliferation of these two types of cancer. Accordingly, miR-30e-5p, miR-378a-3p, miR-302c-3p, miR-520c-3p, miR-10b-5p, and miR-93-5p have tumor-promoting potential because they suppress NK-mediated cytotoxicity, which warrants detailed study.

Furthermore, ovarian cancer tissues exhibit high levels of miR-20a-5p, which downregulates NK cell cytotoxicity in ovarian cancer cells (**Table 3**) and shows tumor-promoting effects (227). Hence, miR-20a-5p overexpression promotes the immune escape of ovarian cancer cells from NK cells. In comparison, miR-106b-5p has a dual role in tumor suppression and in promoting functionality. It is downregulated and upregulated in colon (229) and lung (228) cancer tissues (**Table 3**), respectively, enhancing lung cancer cell proliferation and suppressing colon cancer metastasis.

In contrast, some miRNAs exhibit NK-mediated cytotoxicity (**Table 3**) (**Figure 2**). Classical human leukocyte antigen G (HLA-G) is commonly expressed in renal cancer cells, inhibiting the cytotoxic activity of T and NK cells (230). The overexpression of miR-148a-3p downregulates HLA-G expression and induces cell death in renal cancer cells, activating NK cell cytotoxicity (230) (**Table 3**). Consequently, miR-148a-3p is downregulated in renal cancer cells and, accordingly, possesses a tumor-suppressive function, upregulating NK-mediated cytotoxicity. This warrants a detailed investigation.

### 4.3 Th17 differentiation and CTL function

Some miRNAs, such as miR-326 and miR-181c-5p, induce Th17 differentiation (**Table 3**) (**Figure 2**). Both enhance Th17 development by targeting the negative regulators ETS-1 (231) and SMAD7 (233). miR-326 suppresses lung tumor growth in mice by promoting T cell cytotoxicity (288). It also consistently suppresses immune escape and metastasis in lung cancer cells (288). Th17 cells are crucial for a host’s defense against specific bacteria and fungi and for their anticancer functions (289–291). Accordingly, these miRNAs have anticancer potential. miR-326 is underexpressed in lung, gastric, and breast cancers (232). Similarly, miR-181c-5p shows low expression in cervical (234) and breast (235) cancer cells (**Table 3**). Therefore, miR-326 and miR-181c-5p have anticancer effects, in addition to promoting Th17 differentiation.

Some miRNAs induce CTL cytotoxicity. miR-23a-3p inhibits the cytotoxicity of CD8+ CTLs, while miR-23a-3p knockdown attenuates TGF-β-induced immunosuppression (236) (**Table 3**) (**Figure 2**). Additionally, miR-23a-3p is highly expressed in renal cancer tissues and cells (237), whereas its knockdown inhibits cell proliferation in these cells. Accordingly, miR-23a-3p has regulatory effects on CTL and causes anticancer.

### 4.4 Macrophage M1/M2 polarization and MDSC level

M1 macrophage polarization exhibits pro-inflammatory and anti-tumor functions, whereas M2 macrophage polarization causes immunosuppression and promotes tumor formation (292). Some miRNAs have modulating effects on macrophages (**Table 3**) (**Figure 2**). miR-125b-5p is upregulated in M1 macrophages, enhancing antigen presentation for T-cell activation and inhibiting tumor growth (238). Moreover, miR-125b-5p is underexpressed in breast (239) and liver (240) cancer cells, while their proliferation and migration are inhibited by its overexpression. This suggests miR-125b-5p has tumor-suppressive effects.

In contrast, miR-21-5p and miR-24-2 are downregulated in M1 macrophages. miR-21-5p depletion promotes pro-inflammatory and tumoricidal macrophage (M1) polarization (293) (**Table 3**) (**Figure 2**), while its overexpression inhibits lymphocyte migration and enhances immunotherapeutic resistance to breast cancer (241). M1 macrophage stimulation downregulates miR-24-2 expression, but M2 macrophage stimulation has the opposite effect (294), suggesting that miR-24-2 induces M2 macrophages and promotes tumor growth. Moreover, miR-21-5p and miR-24-2 are highly expressed in oral cancer patients (242) and renal cancer tissues (295) (**Table 3**). Consequently, both have tumor-promoting effects.

Furthermore, MDSCs have immunosuppressive effects (296) and are modulated by miRNAs. miR-17-5p inhibits immune suppression in MDSCs derived from colon tumor-bearing mice (243) (**Table 3**) (**Figure 2**). Moreover, miR-17-5p is highly expressed in gastric cancer patients (244) and, consequently, has tumor-promoting effects.

### 4.5 Potential targets for cytokine-regulating miRNAs

As previously mentioned, target information for these cytokine-modulating miRNAs (**Table 3**) is rarely reported. By applying miRDB data mining (270), their potential targets can be described as follows: miR-150-5p, miR-181a-5p, miR-148a-3p, miR-181c-5p, and miR-23a-3p potentially target HSP90B1; miR-150-5p, miR-181a-5p, and miR-181c-5p can target TNF; and miR-181a-5p and miR-181c-5p potentially target CALR. Moreover, IL12A and IL12B are potentially targeted by miR-21-5p and miR-23a-3p, respectively. In the future, a detailed examination is warranted to explore the role of these targets in miRNA-regulating immune and anticancer activity.

## 5 Relationship between ICD-modulating natural products and miRNA

We analyzed the relationship between ICD-modulating natural products (**Table 1**) and miRNAs (**Tables 2** and **3**). From a miRNA-centric view (**Table 4**), different miRNAs may be regulated by various ICD inducers of natural products; meanwhile, different natural products may regulate the same miRNAs.

**Table 4:** Relationship between ICD-modulating miRNAs and natural products.

ICD-modulating miRNAs	ICD inducers from natural products*	Cell/animal models (natural product effects)
miR-107	doxorubicin (245)	cardiomyocytes (cardiotoxicity)
miR-142-3p	bleomycin (246)	lung epithelial (apoptosis; inflammation)
miR-200a-3p	doxorubicin (247), astaxanthin ↑(248)	cardiotoxicity mice (cardiotoxicity), colon ca (metastasis inhibition)
miR-200c-3p	doxorubicin ↑ (249)	breast ca (apoptosis)
miR-34a-3p	doxorubicin ↑ (250)	cardiomyocytes (cardiotoxicity)
miR-34a-5p	doxorubicin ↑ (251), colchicine (252)	cardiomyocytes (cardiotoxicity), FMF patients
miR-449a	capsaicin (253)	prostate ca (androgen receptor inactivation)
miR-665	bleomycin ↑ (254)	vascular smooth muscle cells (senescence)
miR-223-3p	doxorubicin (204), colchicine (255)	liver ca (autophagy), ACS patients
miR-27b-3p	docosahexaenoic acid (256)	colon ca rat (carcinogenesis)
miR-628-3p	shikonin ↑ (257)	lung ca (apoptosis)
miR-27a-3p	fucoidan (258)	healthy patients
miR-150-5p	doxorubicin (259)	breast ca (antiproliferation)
miR-181a-5p	colchicine ↑ (252)	FMF patients
miR-20a-5p	fucoidan (258)	healthy patients
miR-93-5p	doxorubicin (260), C-phycocyanin ↑ (261), docosahexaenoic acid (256)	cardiomyocytes (apoptosis), lung ca (antiproliferation), colon ca rat (antiproliferation)
miR-106b-5p	doxorubicin (262)	breast ca
miR-326	doxorubicin (263)	breast ca (resistance)
miR-23a-3p	doxorubicin (262)	breast ca
miR-17-5p	doxorubicin (264), shikonin (265), colchicine (255), fucoidan (266)	cardiomyocytes (apoptosis), breast ca (migration inhibition), ACS patients, breast ca (antiproliferation)
miR-21-5p	doxorubicin ↑ (267), docosahexaenoic acid (268)	cardiomyocytes (cardiotoxicity), breast ca (antiproliferation)
miR-24-2	doxorubicin ↑ (269)	heart injury mice (heart injury)

* ↑ indicates that some natural products upregulate their matched miRNAs (left). Except for those indicated with the symbol ↑, the natural products downregulate their matched miRNAs without a symbol. ca, cancer cells; ACS, acute coronary syndrome; FMF, Familial Mediterranean fever.

**Figure 4:**
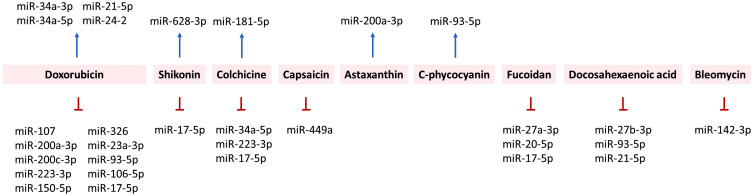
Relationship between ICD inducers of natural products and the regulation of ICD-modulating miRNAs.

From the point of view of natural products, the regulation of miRNAs by doxorubicin, shikonin, colchicine, capsaicin, astaxanthin, C-phycocyanin, fucoidan, docosahexaenoic acid, and bleomycin is summarized in **Figure 4**.

### 5.1 Doxorubicin

In both non-cancer and cancer studies, oxorubicin has exhibited miRNA-modulating effects. In non-cancer studies (cardiomyocytes), doxorubicin has caused cardiotoxicity by upregulating miR-34a-3p (250), miR-34a-5p (251), and miR-21-5p (267). In comparison, doxorubicin downregulated miR-107 (245), miR-93-5p (260), and miR-17-5p (264) in cardiomyocytes. In animal models, doxorubicin induced cardiotoxicity in mice by upregulating miR-24-2 (269) and downregulating miR-200a-3p (247).

In cancer studies, doxorubicin may downregulate or upregulate miRNAs depending on the type of cancer cell. miR-200c-3p suppresses the migration of lung cancer cells by downregulating HMGB1 (146) but enhances the doxorubicin sensitivity of breast cancer cells (249). This finding suggests that doxorubicin may be associated with the upregulation of miR-200c. In comparison, doxorubicin downregulates miR-223-3p in liver cancer cells (204). Under short-term starvation, doxorubicin downregulates miR-23a-3p and miR-106b-5p in breast cancer cells (262). Moreover, miR-150-5p inhibitors suppress the migration and viability of doxorubicin-treated breast cancer cells, suggesting that doxorubicin inhibits breast cancer cell proliferation by downregulating miR-150-5p (259). Accordingly, doxorubicin may differentially regulate miRNAs in various cancer cells.

### 5.2 Shikonin, colchicine, capsaicin, astaxanthin, and C-phycocyanin

Shikonin promotes apoptosis in lung cancer cells by increasing miR-628-3p expression, which is reversed by its inhibition (257). Shikonin inhibits the migration of breast cancer cells by downregulating miR-17-5p, which is overexpressed in breast cancer (265). This suggests shikonin has miR-628-3p-upregulating and miR-17-5p-downregulating abilities.

Colchicine downregulates miR-17-5p and miR-223-3p in acute coronary syndrome (ACS) patients (255). Colchicine upregulates miR-181a-5p but downregulates miR-34a-5p in Familial Mediterranean Fever (FMF) patients (252). Capsaicin upregulates miR-449a in prostate cancer cells (253). Astaxanthin exhibits anti-metastatic effects on colon cancer cells by upregulating miR-200a-3p (248). @Moreover, C-phycocyanin induces miR-93-5p expression in lung cancer cells (297). Accordingly, these miRNAs are differentially regulated by these natural products.

### 5.3 Fucoidan, docosahexaenoic acid, and bleomycin

Fucoidan and docosahexaenoic acid exhibit inhibitory effects on various ICD-modulating miRNAs. For example, fucoidan downregulates human plasma miR-27a-3p and miR-20a-5p in healthy patients (258). It also suppresses the proliferation of breast cancer cells by downregulating miR-17-5p (266). Similarly, docosahexaenoic acid downregulates miR-27b-3p and miR-93-5p in azoxymethane-induced colon cancer in Sprague–Dawley rats (256). Docosahexaenoic acid downregulates miR-21-5p in breast cancer cells (268).

In comparison, bleomycin may exhibit a dual function in regulating ICD-modulating miRNAs. miR-142-3p upregulation attenuates bleomycin-induced injury in lung epithelial cells (246), suggesting that bleomycin downregulates miR-142-3p. In comparison, bleomycin promotes the senescence of vascular smooth muscle cells by upregulating miR-665 (254). Accordingly, these miRNAs are differentially regulated by these natural products.

## 6 Conclusion

ICD is the spatiotemporal immune cell death process caused by the exposure of DAMPs from damaged or dying cancer cells, which triggers the release of many cytokines involved in cancer cell killing. Different DAMPs may be either translocated or show altered secretion in a spatiotemporal manner. However, the functions of DAMPs and cytokines are not limited to the immune response.

DAMPs exhibit intracellular functions but generate extra immunogenic effects when responding to extracellular stimulation (2). Accordingly, they have anticancer and immune-modulating effects. Moreover, natural products and miRNAs have been reported to modulate the immune (DAMPs and cytokines) and cancer cell death responses.

However, there are gaps in the literature regarding the interplay between ICD inducers of natural products and ICD-modulating miRNAs, between natural products and ICD targets, between ICD-modulating miRNAs and ICD targets, and between ICD-modulating miRNAs and immune and cell death responses.

To address these gaps, we have provided an integrated view connecting ICD, cell death responses, miRNAs, and natural products. In this review, we organized reports from the literature regarding the impacts of natural products and miRNAs on the DAMP, cytokine, and cell death responses (apoptosis, autophagy, ferroptosis, necroptosis, and pyroptosis) in various cancer types. We collated and mapped out the potential DAMP and cytokine targets and responses of ICD-modulating miRNAs and natural products. This improvement proves the rationale that ICD inducers of natural products modulate miRNAs, and they, in turn, target DAMPs and cytokines, triggering immune and cancer cell death responses.

Notably, we used miRDB to process target retrieval. This is a comprehensive and reliable database constituting a vast array of experimental data, but the target information acquired may be derived from different cells. This target information needs to be further validated in future ICD studies. Moreover, the functions of DAMPs and miRNAs are context-dependent (2). For instance, HMGB1 exhibits pro- and anti-tumoral functions depending on its location in extracellular or intracellular environments. Besides its immune functions, HMGB1 exhibits tumor suppression and oncogenic functions in the context of receptors, targeted cells, and redox status (298). Moreover, miRNAs can regulate different immune responses and modulate death responses in cancer cells. A detailed investigation of the relationship between the natural products and miRNA-modulated immune and cell death responses needs to be conducted in the future.

Altogether, this review summarizes the changes in DAMPs and cytokines and cell death responses in cancer cells, linking these with natural products and miRNAs with ICD-modulating effects. This work sheds light on the anticancer effects of natural products and the mechanisms by which they modulate ICD with respect to miRNAs.
